# Analysis of Uterine Blood Flow in Breeding Sows through the Estrus and Early Diestrus, and after Artificial Insemination

**DOI:** 10.3390/vetsci9060260

**Published:** 2022-05-30

**Authors:** Salvador Ruiz, Juan Carlos Gardón, Iván Hernández-Caravaca, Chiara Luongo, Francisco Alberto García-Vázquez

**Affiliations:** 1 Departamento de Fisiología, Facultad de Veterinaria, Campus de Excelencia Mare Nostrum Universidad de Murcia, 30100 Murcia, Spain; sruiz@um.es (S.R.); chiara.luongo@um.es (C.L.); 2 Instituto Murciano de Investigación Biosanitaria Hospital Virgen de la Arrixaca (IMIB-Virgen de la Arrixaca), 30120 Murcia, Spain; ivan.hernandez@ua.es; 3 Department of Animal Medicine and Surgery, Faculty of Veterinary and Experimental Sciences, Catholic University of Valencia-San Vicente Mártir, Guillem de Castro 106, 46003 Valencia, Spain; jc.gardon@ucv.es; 4 Departamento de Enfermería Comunitaria, Medicina Preventiva y Salud Pública e Historia de la Ciencia, Facultad Ciencias de la Salud, Campus de Sant Vicent del Raspeig, E-03080 Sant Vicent del Raspeig, Spain

**Keywords:** arterial blood flow, imaging, Doppler ultrasound, echography, porcine

## Abstract

This study aimed to determine uterine blood flow indices by transabdominal Doppler ultrasound in sows (*n* = 18) under different conditions: (i) sows after estrus detection (day 0, D0); (ii) sows 2 h after artificial insemination (AI), performed 24 h after detection of estrus (day 1, D1); (iii) sows in early diestrus (day 5, D5). Moreover, three different types of seminal doses were used for AI depending on the ejaculate fraction included (F1: doses containing only the rich fraction of the ejaculate; F2: F1 + the transition fraction between rich and poor fractions; F3: F2 and poor fraction). The statistical analysis revealed significant differences in some indices regarding the period of analysis (D0, D1, and D5). Diastolic velocity and mean velocity showed lower values at D5 in comparison with D0 and D1 (*p* < 0.01). On the other hand, the pulsatility index and the relationship systolic velocity/diastolic velocity indicated higher values at D5 in comparison with D0 and D1 (*p* < 0.01). No differences were observed regarding the type of seminal dose used in any of the time points analyzed (*p* > 0.05). Neither insemination per se nor the type of ejaculate fraction used immediately modified the uterine vascularity, but some indices are affected by the stage of the estrus cycle (estrus vs. early diestrus).

## 1. Introduction

In sows, the use of real-time ultrasonography is a fast, practical, and accurate tool for the diagnosis of different reproductive processes. Thus, transabdominal B-mode ultrasonography has now been recognized as a routine technique for a multitude of purposes, including pregnancy testing, puberty monitoring, or assessment of follicular dynamics, among other clinical uses [1,2,3,4]. Moreover, in veterinary medicine, Doppler ultrasound has become a common non-invasive method to study the perfusion characteristics of the female reproductive tract. Doppler mode allows for the quantitative and qualitative evaluation of the irrigation of organs and can be used as an important diagnostic technique for evaluating uterine irrigation and function [5]. In fact, several studies have evidenced, through Doppler ultrasonography, the relationship between blood flow with ovarian and uterine function throughout the estrus cycle and pregnancy [6].

Different Doppler methods, such as color, power, and pulse wave Doppler, are feasible for reproductive purposes. There are different reports of evaluation of the characteristics of uterine perfusion using Doppler through transabdominal exploration in bitches [7] or transrectal in cows and mares [8,9]. In the case of pigs, there are some reports in which invasive methods were used [10,11,12,13,14,15,16,17,18,19]. These methods require special working conditions such as surgical implantation of the probe and pre- and post-surgical care of the animals, and the animals must be placed in adapted crates to adequately monitor the flow during the assessment. However, the use of non-invasive methods in this species is scarce [20,21,22,23]. In any case, the estrus cycle influenced by hormone status seems to have an effect on uterine blood flow. In fact, Dickson et al. (1969) [22] demonstrated that estrogen administration exerts an increase in blood flow in the porcine uterus. Similarly, Ford et al. (1982) [12] reported that both estradiol-17β and estrone concentrations in the uterine fluid were significantly different in pregnant compared to non-pregnant sows. More recently, it has been reported that uterine blood flow characteristics show specific patterns throughout the estrus cycle [20]. Specifically, uterine perfusion is highest in the proestrus phase compared to estrus and remains low in metestrus and most of diestrus. Furthermore, Krzymowski and Stefańczyk-Krzymowska (2002) [23] described a new theory that states that estrogens and progesterone and other factors such as PGE2, LH, oxytocin, cytokines, and neurotransmitters are the main factors that alter blood flow at the uterine level. Likewise, during the preovulatory period, there is a high concentration of estrogen and LH, as well as a low level of progesterone in the blood. This ratio of reproductive hormones causes dilation of the uterine artery and all its blood vessels. Therefore, during proestrus, estrus, ovulation, and the following three to four days, the blood supply to the uterus is maximal [24,25,26]. During early diestrus (day 4 to 5), progesterone levels begin to predominate over estradiol. Therefore, an increase in uterine weight [27] and a decrease in blood flow through the uterine artery are observed during this phase [11].

The estrus stage is one of the keys to swine production because it is the period where the AI is carried out. The AI process does not only consist of the deposition of semen in the female genital tract. A new paradigm is becoming evident that demonstrates the importance of semen transit modulating the female reproductive tract at the time of insemination. Seminal plasma infusion into the porcine uterus induces significant cellular inflammation 36 h after infusion that was still evident 8 days later, including an increase in uterus vascularity [28]. However, this process has been only demonstrated by visual observation of the genital tract [28] but not by the Doppler methodology. Moreover, the effect of the seminal plasma on the female genital tract also depends on the fraction of the ejaculate included [29]. Thus, the effect of different ejaculate parts on the blood flow uterine environment has yet to be elucidated.

From the above information, we hypothesized that the measurement of blood flow parameters of sows by transabdominal Doppler ultrasound may help to better understand the dynamics of the uterus during estrus, early diestrus, and due to AI. Thus, the present work aimed to determine uterine blood flow indices under different conditions: (i) sows after estrus detection (day 0, D0); (ii) sows 2 h post-AI, performed 24 h after detection of estrus (day 1, D1); (iii) sows in early diestrus (day 5, D5). Moreover, the AIs were performed using different fractions of the ejaculate, and sows were divided according to the type of fraction used for AI (F1: rich fraction of the ejaculate; F2: F1 + intermediate fraction; F3: F2 + poor fraction).

## 2. Material and Methods

### 2.1. Ethics

All procedures for this study were approved by the Ethical Committee of the University of Murcia on 1 June 2020 (PID2019-106380RB-I00). Through the experiments, animals were handled carefully avoiding any unnecessary stress. All experiments were performed following relevant guidelines and regulations. The study was carried out in compliance with the ARRIVE guidelines (https://arriveguidelines.org/, accessed on 20 April 2021).

### 2.2. Animals and Study Design

A total of 6 fertility proved boars (Pietrain German Genetics; 35.6 ± 7.5 months of age) were used for the experiment. Boars were housed in individual pens (according to the European Commission Directive for Pig Welfare) with sawdust in a commercial boar stud (Sergal Gestió Ramadera, Lleida, Spain). Temperature levels were controlled automatically by a climate control system that maintained the temperature in the room between 18 and 22 °C. Boars had a restricted feeding regime according to their nutritional requirements, while water was available *ad libitum*.

A total of 18 crossbred sows (Large-White X Landrace, Danbred genetic) from a commercial sow farm were used for this study (Genera S.L., Lorca, Spain). At weaning, sows were selected by parity (from 3 to 6; mean parity of 4.22 ± 0.87) and weaning-to-estrus interval (from 3 to 4; mean of 3.77 ± 0.42 days) (Table 1).

**Figure 1:**
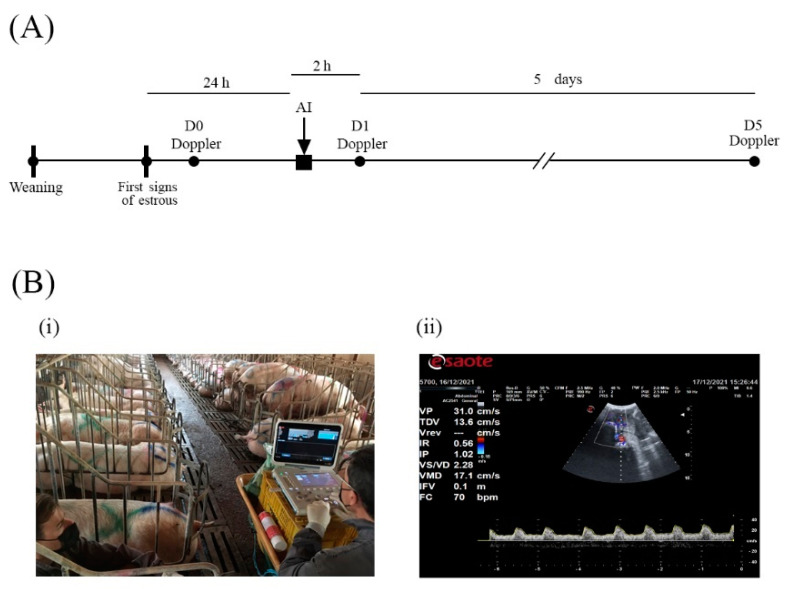
(**A**) Timeline of the procedures carried out during the study. Black dots (D0, D1, and D5) indicate the moment when Doppler ultrasound analysis was performed. The black square indicates the moment of artificial insemination (AI). (**B-i**) Detail of the procedure of transabdominal ultrasonography carried out during the analysis. The assay was performed by two researchers, one in charge of the ultrasound probe, while the other was analyzing the real-time images. (**B-ii**) A representative image was displayed by the ultrasound apparatus (MyLab^TM^ alpha, Esaote España S.A., Barcelona, Spain) during the evaluation of the uterine arteries of a sow.

Doppler ultrasound assays were performed in sows at three different conditions (Figure 1a). The first one was carried out after estrus detection (D0). Twenty-four hours after the onset of the estrus, the females were artificially inseminated, and 2 h later, the ultrasonography assay was performed (D1). For the AI, three types of seminal doses, differentiated by the ejaculate fraction included (F1, F2, and F3), were used. Finally, 5 days after the onset of estrus, the same procedure was conducted (D5).

### 2.3. Semen Collection

A total of 6 ejaculates were collected in a pre-warmed thermal cup by the gloved hand method. The ejaculates were collected according to the fractions included: (1) F1 (one fraction): ejaculate containing only the rich fraction (*n* = 2). This fraction is characterized by a dense white color. (2) F2 (two fractions): includes F1 and the transition fraction between rich and poor fractions, which consists of a less dense white color of the ejaculate rich fraction (*n* = 2). (3) F3 (three fractions): includes F2 and poor fraction characterized for a water-like liquid aspect (*n* = 2). For each collected ejaculate, independently of the fraction/s included, the pre-sperm phase of the ejaculate was discarded, and the gel fraction was removed using a filter. During the trial, the semen collection was always carried out by the same technician.

### 2.4. Seminal Dose Preparation and Conservation 

The semen, once obtained, was diluted in an AndroStar^®^ Plus extender (Minitüb, Tiefenbach, Germany), and the concentration was adjusted to ≈33 × 10^6^ sperm/mL. Sperm concentration was calculated using an automatic sperm analyzer (Androvision^®^ Minitüb, Tiefenbach, Germany). Then, semen was packaged in plastic bags (2000 × 10^6^ sperm/60 mL) and color-labeled depending on the type of seminal doses for better identification at the lab and farm (F1 = white color label; F2 = blue color; F3 = pink color). The semen preparation was carried out by the same technician during the period of the trial. Finally, seminal doses were kept at refrigeration (≈16 °C) until they were used for semen evaluation and AI.

### 2.5. Sperm Analysis

The sperm quality from seminal doses was evaluated before AI. The following sperm parameters were assessed:

#### 2.5.1. Motility Analysis by CASA

Sperm motility was analyzed by Computer-Assisted Semen Analysis (CASA; ISAS^®^ software, PROiSER R+D S.L., Valencia, Spain) coupled with phase-contrast microscopy (negative-pH 10x objective; Leica DMR, Wetzlar, Germany) and a digital camera (Basler Vision, Ahrensburg, Germany). An aliquot from each sample was warmed at 38 °C for 10 min and a 4 µL drop was placed in a pre-warmed (38 °C) chamber (20-micron Spermtrack^®^ chamber, Proiser R+D, SL; Paterna, Spain) and evaluated. CASA setting parameters were adjusted to 25 frames per second and particle size area between 10 and 80 mm^2^. Sperm cells were considered to be motile with an average path velocity >10 μm/s, and progressively motile with a straightness >45%. At least 3 fields per sample were recorded and evaluated.

#### 2.5.2. Viability and Acrosome Status Assay

Sperm viability and acrosome status were evaluated by a staining solution prepared with 50 µL of propidium iodide (PI) 500 µg/mL (P-4170 Sigma-Aldrich^®^, Madrid, Spain) and 100 µL of Arachis hypogea lectin PNA-FITC 200 µg/mL (Sigma-Aldrich^®^, Madrid, Spain) in 10 mL of PBS without calcium and magnesium (Sigma-Aldrich^®^, Madrid, Spain). Sperm samples were incubated with PI/PNA-FITC solution for 10 min at room temperature in the dark. Then, sperm cells were observed in transmitted light bright field and fluorescence microscopy (Leica^®^ DM4000 Led, Wetzlar, Germany, 495/520 nm). At least 200 sperm per sample were evaluated. Spermatozoa without fluorescence were classified as live and with intact acrosome, sperm showing red fluorescence were classified as dead, and sperm with green fluorescence were classified as sperm with damaged acrosome.

#### 2.5.3. Mitochondrial Activity

Sperm mitochondrial activity was evaluated by a staining solution prepared with 10 µL of JC-1 0.017 µg/mL (5,5′,6,6′-tetrachloro-1,1′,3,3′-tetraethylbenzimidazolylcarbocyanine iodide; ThermoFisher Scientific Inc., Waltham, MA, USA) in 10 mL of PBS without calcium and magnesium. Sperm samples were incubated with JC-1 solution for 30 min at 38 °C in the dark. Then, sperm cells were observed under fluorescence microscopy (Leica^®^ DM4000 Led, Wetzlar, Germany, 495/520 nm). At least 200 sperm per sample were evaluated. Spermatozoa with orange fluorescence were classified as sperm with high mitochondrial membrane potential, and sperm with green fluorescence were classified as sperm with low mitochondrial membrane potential.

### 2.6. Doppler Ultrasound

An ultrasound scanner (MyLab^TM^ alpha, Esaote España S.A., Barcelona, Spain) equipped with a 2–7 MHz abdominal sector probe and Doppler measurement recording software was used for scanning animals. The settings frequency (2–2.5 MHz), depth (170 mm), and pulse repetition frequency (1–2.5 KHz) were standardized and remained constant for all examinations. Each recording was made for a minimum period of 6 s and with at least 5 valid waves. At least 3 valid records were made for the animal in each measurement point (D0, D1, and D5). The animals were housed in their insemination crates, and to prevent their movements from interfering with an adequate performance of the ultrasound recording, the ultrasound assessments were performed when the animals were lying down and calm (see Figure 1(B-i)). The scanning procedure lasted for approximately 20 min per sow.

In the Doppler velocimetry assessment of arteries, firstly, two-dimensional color flow mode Doppler scans must be initially performed to visualize the vessels at the desired location. Secondly, blood vessels and flow must be identified using pulsed waved Doppler. Red indicates blood flow towards the transducer and blue indicates blood flow away from the transducer [30]. Before the assessment of Doppler parameters of the arteries, a clear differentiation between the arteries and veins should be conducted. To differentiate them, spectral Doppler should be performed, wherein the artery will typically have a spectral waveform representing the arterial pulse in each cardiac cycle (see Figure 1(B-ii)). In the vein, however, the flow has no pulse, and it is almost constant. Once the uterine myometrial arterial plexus was located, the Doppler caliper was placed in the lumen of the vessel, and arterial blood flow was plotted as a waveform.

The blood flow was evaluated in an individual vessel, and semi-quantitatively Doppler indices were recorded. These indices have been used to obtain information about blood flow and vascular impedance that cannot be obtained from velocity information alone. They depend on the measurements of the *systolic velocity* (SV)*, diastolic velocity* (DV)*,* and *mean velocity* (MV). The two widely used indexes are the *Resistance Index* (RI) and *Pulsatility Index* (PI) [31]. They are not a direct measure of blood flow, but rather describe the resistance to blood flow in vessels peripheral to the vessel being examined. *Resistance Index* (RI) indirectly reflects the resistance that the blood flow presents to circulate through the vascular lumen and assumes the maximum value of the blood flow, so it does not allow differentiation with the final diastolic flow that reaches values from zero. This index is suitable for those vessels in which blood flow persists during diastole. The RI is calculated using the formula RI = (SV − DV)/SV. *Pulsatility Index* (PI) refers to the speed of blood flow and is used in those tissues with high vascular resistance, in which there is a backflow of blood during diastole. The PI measures the total distance from the top to the bottom of the systolic peak and divides this by the mean velocity over the cardiac cycle. It is expressed as PI = (SV − DV)/MV. It is suitable for those vessels where flow is absent during diastole [5].

The ultrasound machine displays the record flows as a spectral curve. The area within this spectral curve is calculated by the software of the scanner. This area is referred to as the FVI (*flow velocity integral*), and it measures how far blood travels during the flow period. Finally, *heart rate* (HR) in beats/minute, was recorded during Doppler ultrasound assays. The parameters evaluated by Doppler during the study are described in Table 2.

### 2.7. Estrus Detection and Artificial Insemination (AI) 

Multiparous sows were weaned 28 days after farrowing. Estrus detection, starting on the day of weaning, was performed twice daily (in the morning and the afternoon) by the same experienced workers, allowing sows nose-to-nose contact with a sexually mature boar and applying backpressure. Sows exhibiting vulva reddening and swelling, and a standing reflex were considered in estrus. After estrus detection, sows were randomly assigned to one of the treatment groups (F1, F2, or F3). Sows were inseminated in individual crates by the post-AI method (as previously described by Hernández-Caravaca et al., 2012) [32] 24 h after the onset of estrus and 24 h later, using a combined catheter-cannula kit (Soft & Quick^®^, Tecno-Vet, S.L., Barcelona, Spain).

### 2.8. Return to Estrus and Pregnancy Diagnosis

The return to estrus was evaluated using male sexual stimulation and applying back pressure from days 18 to 24 after insemination in search of standing reflex. Furthermore, pregnancy was confirmed by ultrasound 23–28 days after insemination by transabdominal ultrasonography (Echoscan T-300 S, Barcelona, Spain). Abortions were monitored by the technician’s direct visualization and confirmed by ultrasonography.

### 2.9. Farrowing and Litter Performance

At 110 days of gestation, pregnant sows were moved from the gestation facilities to the farrowing pens. At the end of farrowing, fertility (%), farrowing (%), the total number of piglets born, and the number of piglets born alive were recorded. Moreover, the fecundity index was calculated by multiplying the farrowing rate by the number of live-born piglets.

### 2.10. Statistical Analysis

Data were analyzed using the IBM SPSS 24 Statistics package (SPSS, Chicago, IL, USA) and Statistic Analysis Software (SAS, University Edition 2016). For data comparison between time points (D0, D1, D5), the assumption of normality was checked by the Shapiro–Wilk test. Data that were not normally distributed (SV, MV, SV/DV, RI, PI, FVI, HR) were tested by the Kruskal–Wallis test. When the assumption of normality was confirmed (DV), data were analyzed by ANOVA with the post hoc Tukey test. For the evaluation of the effect of ejaculate fractions (F1, F2, and F3) on Doppler ultrasonography parameters in sows during the different time points (D0, D1, and D5), sphericity for repeated measurements was assessed using the restricted likelihood ratio test Huynh–Feldt (H-F) and Greenhouse–Geisser (G-G) covariance structures. If the difference between H-F and G-G tests (distributed under the null hypothesis as a χ2 with the difference between the degrees of freedom, df) was greater than χ2 df, the sphericity of the data was considered. All the variables confirmed the sphericity of the data, and they were analyzed using Proc Mixed procedures. The model included the treatments (F1, F2, and F3), the three days of evaluation (D0, D1, and D5), and the interaction between these as the main effect, with different samples and sows as the random effect. A first-order autoregressive covariance structure was used to adjust the difference in data according to the differences with time, and the Tukey post-hoc test was applied to detect differences between experimental groups. Data were expressed as mean ± standard error of the mean (SEM), and differences were considered statistically significant when *p* < 0.05.

## 3. Results

**Figure 2:**
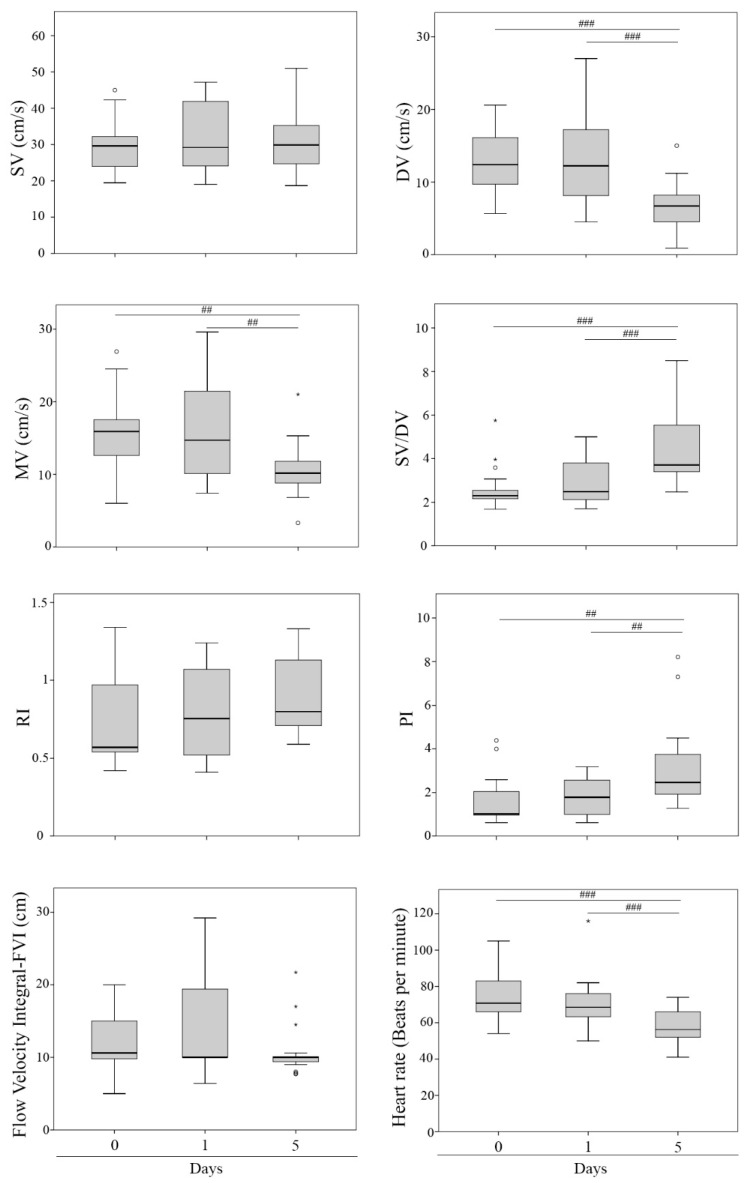
Box plot describing Doppler ultrasonography values of the uterine arteries’ blood flow at the three days of evaluation (D0, D1, and D5). Significant differences between experimental groups are indicated as ## (*p* < 0.01) and ### (*p* < 0.001). Small circles (°) and asterisks (*) in the box plot represent outliers and extreme cases, respectively.

**Figure 3:**
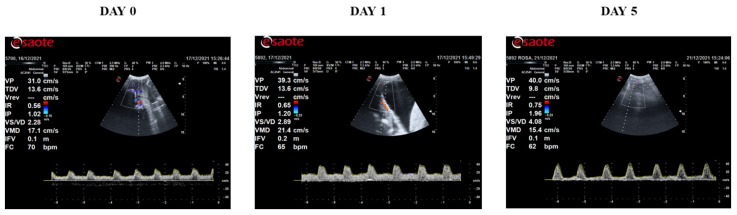
Demonstrative waveform pulse–Doppler representing variation in the blood flow of uterine arteries indices measured during estrus (D 0) and after AI (D1 and D5) using Doppler ultrasonography in three selected sows. Representative Doppler ultrasound images were obtained using the MyLab^TM^ alpha (Esaote España S.A., Barcelona, Spain) color Doppler ultrasound machine.

**Figure 4:**
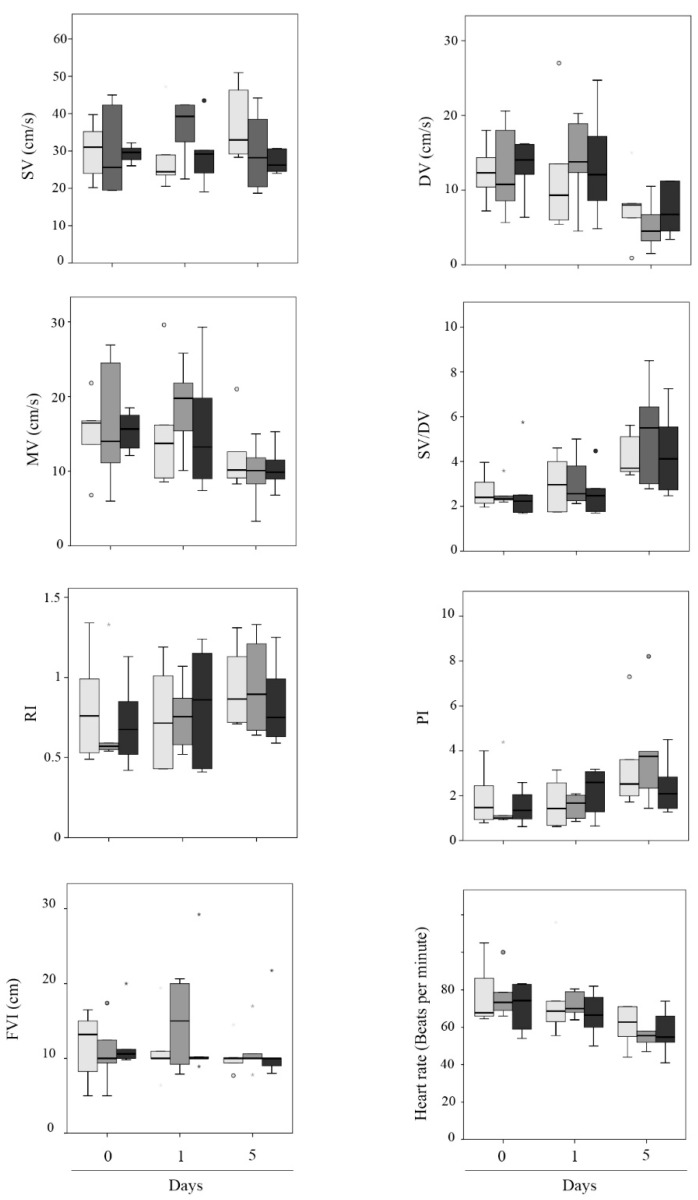
Box plot describing Doppler ultrasonography values of the uterine arteries’ blood flow at the three days of evaluation (D0, D1, and D5) analyzed by the type of seminal dose used (F1—light grey bars, F2—grey bars, and F3—dark grey bars). The statistical comparison was performed between seminal dose types (F1-F2-F3) on each day of analysis (D0, D1, and D5). No significant differences were observed (*p* > 0.05). Small circles (°) and asterisks (*) in the box plot represent outliers and extreme cases, respectively.

Some Doppler indices displayed differences over time (Figure 2), showing similarities between D0 and D1 but differences in D5 (Figure 3). The DV values decreased twofold from D0 (12.65 ± 1.03 cm/s) and D1 (12.97 ± 1.60 cm/s) to D5 (6.73 ± 0.89 cm/s) (*p* = 0.002 and *p* = 0.001, respectively). Moreover, the MV showed a decrease from D0 (15.60 ± 1.30 cm/s) and D1 (16.42 ± 1.69 cm/s) to D5 (10.68 ± 0.92 cm/s) (*p* = 0.027 and *p* = 0.008, respectively). Regarding SV/DV, it increased from D0 (2.62 ± 0.24) and D1 (2.89 ± 0.26) to D5 (4.56 ± 0.42) (*p* < 0.001 and *p* = 0.001, respectively). Furthermore, PI increased from D0 (1.63 ± 0.27) and D1 (1.76 ± 0.22) to D5 (3.15 ± 0.48) (*p* = 0.007 and *p* = 0.015, respectively). Moreover, HR was higher at D0 (74.72 ± 3.21 bpm) and D1 (71.02 ± 3.42 bpm) than D5 (57.50 ± 2.25 bpm) (*p* < 0.001 and *p* = 0.005, respectively). The rest of the parameters (FVI, RI, and SV) did not show any significant difference over time (Figure 2). Before AI, sperm quality in the seminal doses was analyzed, showing no differences between experimental groups (F1, F2, F3) in any parameters (Table 3). When the Doppler indices were compared between the fraction/s included in the seminal doses (F1, F2, F3) at each time point, no significant differences were observed (Table 4, Figure 4). Additionally, no interaction between treatments and time was found for any parameter. The reproductive outcomes of the sows used in the study were similar between groups (Table 5).

## 4. Discussion

The swine industry is constantly looking for an increase in the pigs’ production efficiency, with the reproductive output being crucial for this purpose. In this respect, the estrus and the insemination stages play a key role. Therefore, the evaluation of the uterine blood flow indices to increase the knowledge about uterine changes during the estrus cycle and after AI could be of interest to the swine sector. For this reason, in the present study, a non-invasive method, the transabdominal Doppler ultrasound, was used to investigate the uterine blood flow parameters in sows during different periods of the estrus cycle (estrus before AI, estrus after AI, early diestrus). Moreover, the AI was performed with seminal doses prepared from different ejaculate fractions. From the results obtained, several blood flow parameters changed during the estrus cycle, but they are not influenced by the type of ejaculate fraction used for AI.

Changes in uterine perfusion throughout the estrus cycle in pigs have been reported previously from research on gilts at different stages of the estrus cycle [20,33,34,35]. Changes to the uterine architecture including perfusion characteristics in pigs are known to be hormone dependent [34,36]. The time points selected for our analysis coincide with different levels of sexual hormones, presenting high levels of estradiol and low level of progesterone in D0 and D1, and vice versa for D5 [37]. High concentrations of estrogens in proestrus and partially also in estrus lead to vasodilation [11]. Estrogens are involved in the expression of genes that synthesize vasodilator substances such as prostacyclin and nitric oxide and thus high blood supply [38]. In contrast, progesterone leads to vasoconstriction and thus to low blood supply [11]. Our results have indicated a variation in some Doppler indices during time points analysis (D0, D1, and D5), showing a decrease in MV indices on D5 (early diestrus) compared to D0 and D1 (estrus). These results agree with a previous report [20], conducted in gilts where low mean blood flow values were obtained during estrus, metestrus, and early and mid-estrus compared to other stages of the estrus cycle. It has been previously shown [11] that uterine blood flow in sows increases from day -5 (day 0 = estrus) towards estrus and declines throughout diestrus. Estradiol concentrations in the ovulating cows have also positively correlated with the increase in uterine arteries’ blood flow velocities [39]. Likewise, an increase in uterine blood flow has been demonstrated as a consequence of estrogen administration in sows [33], ewes [40,41,42], and cows [43]. In this regard, it has been reported that these blood flow changes may be caused by steroids of ovarian origin that modify the function of the uterine periarterial sympathetic system by altering the number of α1-adrenergic receptors [26] or other associated factors such as prostaglandins, LH, oxytocin, or cytokines [23]. Therefore, these changes in blood flow at the uterine level in different animal species are associated with the estrogen–progesterone ratio in the systemic circulation and its effects at different physiological levels [26]. Therefore, we assume that the oestrogenic phase of the estrus cycle modifies the uterine blood flow in sows.

Regarding changes in blood flow parameters during the estrus cycle, PI values increased from day D0 and D1 to D5, which agrees with the work of Hertl et al. (2018) [20], who obtained mean uterine PI values that started to increase slowly during estrus and metestrus to then rise significantly in the first part of the diestrus. Decreased PI values indicate a reduction in resistance to blood flow and in turn increased arterial perfusion and continuous supply of oxygen and nutrients to the myometrium [44]. In contrast, the values recorded for RI remain without significant changes in the periods of estrus and early diestrus analyzed, as reported previously [20]. Finally, heart range values obtained were according to previous results in pigs [45]. 

When sperm is deposited in the female genital tract after the IA, an interaction between the components of the seminal dose (sperm, seminal plasma, and extender) and uterine environment (epithelial cells, blood cells, and uterine fluid) starts. The response of the uterus to insemination is a transitory inflammation in the form of an influx of polymorphonuclear neutrophilic granulocytes (PMN) into the uterine lumen [46,47], in which seminal plasma seems to modulate the uterine inflammatory response. Moreover, seminal plasma contains prostaglandins and estrogen, which have vasodilatory features [26,48] able to affect the blood flow. In fact, and contrary to our initial hypothesis, there was no effect of insemination on uterine blood flow. One of the reasons for this result may be explained by the composition of the seminal dose. In our case, the seminal dose is composed of sperm, seminal plasma, and extender. Thus, the putative effect of the seminal plasma on the uterine blood flow could have been mitigated by the low percentage of this component on the dose type. Even more, there were no effects on seminal dose type (different proportion and composition of seminal plasma F1 = 7.91 ± 3.76; F2 = 9.15 ± 2.92; F3 = 15.19 ± 7.42% of seminal plasma [49]) on uterine blood flow after insemination. In contrast, a significant increase in uterine perfusion had been observed in mares but after infusion of seminal plasma or raw sperm. In fact, this effect was prolonged until 24 h in the case of the seminal plasma but only 1h for raw sperm [50]. Another aspect to have in consideration is that the uterine blood flow was not measured just before and after AI. However, progesterone was analyzed in the sows at different time points (D0, D1, and D5) in order to verify the estrus stage of the sows included in our study. Progesterone showed similar levels at D0 and D1 (0.27 ± 0.06 and 0.52 ± 0.28 ng/mL, respectively), assuming that those time points have similar behavior. However, progesterone levels were significantly higher (15.56 ± 13.89 ng/mL; *p* < 0.05) at D5 in comparison with D0 and D1.

All of the animals used during the study had similar reproductive performance in terms of fertility and litter size, so no comparison may be made between animals of different outputs. Actually, changes in uterine blood flow have been associated with endometrial changes throughout the post-breeding period in mares [9] and endometritis in dairy cows [51], which has the potential for determining fertility. Further studies could then be conducted to determine whether fertility and prolificacy failures could be associated with changes in uterine blood flow around the period of estrus and insemination in swine.

## 5. Conclusions

In conclusion, this study showed that uterine blood flow indices can be measured effectively by the non-invasive method of transabdominal Doppler ultrasound. Our findings demonstrated how some parameters such as diastolic velocity, mean velocity, systolic velocity/diastolic velocity, pulsatility index, and heart rate changed during the estrus cycle (estrus vs. early diestrus). However, the AI and the type of seminal doses used did not affect the uterine vascularity, with seminal plasma highly being diluted, and sperm quality being similar between the fractions. In line with these findings, reproductive performance was also not affected by uterine perfusion. 

## Figures and Tables

**Table 1:** Parity and weaning-to-estrus interval of the sows used in the experiment. Data are represented as mean ± SD (standard deviation). The global group includes the data of all the sows used in the study.

Experimental Group	Sows (n)	Parity	Weaning-To-Estrus Interval (Days)
Global	18	4.22 ± 0.87	3.77 ± 0.42
F1	6	4.33 ± 1.03	3.83 ± 0.40
F2	6	4.16 ± 0.75	3.83 ± 0.40
F3	6	3.92 ± 0.98	3.66 ± 0.51

**Table 2:** Description of Doppler ultrasound indices.

Doppler Ultrasound Parameters	Abbreviation	Units	Description
Systolic velocity	**SV**	cm/s	Peak systolic velocity
Diastolic velocity	**DV**	cm/s	End diastolic velocity
Mean velocity	**MV**	cm/s	Mean velocity
Systolic velocity/diastolic velocity	**SV/DV**	-	SV/DV relationship
Resistance Index	**RI**	-	(SV − DV)/SV
Pulsatility Index	**PI**	-	(SV − DV)/MV
Flow velocity integral	**FVI**	cm	Area within the spectral curve
Heart rate	**HR**	bpm	Beats per minute

**Table 3:** Spermatozoa quality values from different seminal dose types used (F1, F2, and F3) and global values (including F1, F2, and F3). Data are represented as mean ± SEM (standard error of the mean).

	Total Motility (%)	Progressive Motility (%)	Viability (%)	Acrosome Integrity (%)	Mitochondrial Activity (%)
Global	90.5 ± 2.7	51.6 ± 6.0	94.0 ± 0.6	97.0 ± 0.2	91.6 ± 1.0
F1	93.0 ± 1.0	49.5 ± 14.5	93.5 ± 1.5	97.0 ± 0.0	92.5 ± 1.5
F2	87.5 ± 8.5	53.5 ± 6.5	94.0 ± 1.0	97.0 ± 0.0	91.5 ± 1.5
F3	91.5 ± 3.1	52.0 ± 17.0	94.5 ± 1.5	97.0 ± 1.0	91.0 ± 3.0

**Table 4:** Effect of ejaculate fraction (F1, F2, or F3) on Doppler ultrasonography values of the uterine arteries blood flow. Data include the statistical analysis of repeated measures (D0, D1, and D5). Data are shown as the mean ± SEM.

	F1	F2	F3	*p*-Value
SV (cm/s)	31.73 ± 2.23	31.88 ± 2.40	28.52 ± 1.21	0.54
DV (cm/s)	10.64 ± 1.41	10.49 ± 1.49	11.22 ± 1.32	0.93
MV (cm/s)	14.12 ± 1.41	14.88 ± 1.68	13.71 ± 1.32	0.89
SV/DV	3.28 ± 0.28	3.51 ± 0.44	3.22 ± 0.40	0.69
RI	0.83 ± 0.07	0.80 ± 0.07	0.79 ± 0.07	0.95
PI	2.26 ± 0.39	2.26 ± 0.46	1.99 ± 0.25	0.81
FVI (cm)	11.09 ± 0.89	12.08 ± 1.15	12.18 ± 1.45	0.84
HR (bpm)	70.52 ± 4.17	67.66 ± 3.07	65.07 ± 3.09	0.67

**Table 5:** Pregnancy rate (%), farrowing rate (%), total-born piglets, live-born piglets, and ± fecundity index (mean ± standard deviation) in sows inseminated with the three experimental groups (F1, F2, F3). The global group includes the data of all the sows used in the study.

Experimental Group	Sows (n)	Pregnancy Rate (%)	Farrowing Rate (%)	Total Born Piglets (n)	Live-Born Piglets (n)	Fecundity Index * (n)
Global	18	100	94.4	20.17 ± 4.6	15.58 ± 4.7	1471.5 ± 450.3
F1	6	100	100	22.66 ± 4.4	14.50 ± 5.6	1450.0 ± 568.3
F2	6	100	83.3	20.60 ± 1.6	18.20 ± 2.8	1516.0 ± 238.5
F3	6	100	100	17.33 ± 5.5	14.50 ± 4.9	1450.0 ± 92.9

* Fecundity index was calculated as follows: farrowing rate multiplied by the number of live-born piglets.

## Data Availability

The original contributions presented in the study are included in the article, and further inquiries can be directed to the corresponding author.
